# Maintaining quality of health services after abolition of user fees: A Uganda case study

**DOI:** 10.1186/1472-6963-8-102

**Published:** 2008-05-09

**Authors:** Juliet Nabyonga-Orem, Humphrey Karamagi, Lynn Atuyambe, Fred Bagenda, Sam A Okuonzi, Oladapo Walker

**Affiliations:** 1 Health systems unit, World Health Organization, Kampala, Uganda; 2 Health systems unit, World Health Organization, Nairobi, Kenya; 3 Department of community health and behavioral science; School of public health – Makerere University; Kampala, Uganda; 4 Department of community medicine, Mbarara University of Science and Technology, Mbarara, Uganda; 5 Regional Centre for Quality of Health Care; School of public health – Makerere University; Kampala, Uganda; 6 Department of Technical cooperation, World Health Organization – Regional office for Africa, Brazzaville, Republic of the Congo

## Abstract

**Background:**

It has been argued that quality improvements that result from user charges reduce their negative impact on utilization especially of the poor. In Uganda, because there was no concrete evidence for improvements in quality of care following the introduction of user charges, the government abolished user fees in all public health units on 1^st ^March 2001. This gave us the opportunity to prospectively study how different aspects of quality of care change, as a country changes its health financing options from user charges to free services, in a developing country setting. The outcome of the study may then provide insights into policy actions to maintain quality of care following removal of user fees.

**Methods:**

A population cohort and representative health facilities were studied longitudinally over 3 years after the abolition of user fees. Quantitative and qualitative methods were used to obtain data. Parameters evaluated in relation to quality of care included availability of drugs and supplies and; health worker variables.

**Results:**

Different quality variables assessed showed that interventions that were put in place were able to maintain, or improve the technical quality of services. There were significant increases in utilization of services, average drug quantities and stock out days improved, and communities reported health workers to be hardworking, good and dedicated to their work to mention but a few. Communities were more appreciative of the services, though expectations were lower. However, health workers felt they were not adequately motivated given the increased workload.

**Conclusion:**

The levels of technical quality of care attained in a system with user fees can be maintained, or even improved without the fees through adoption of basic, sustainable system modifications that are within the reach of developing countries. However, a trade-off between residual perceptions of reduced service quality, and the welfare gains from removal of user fees should guide such a policy change.

## Background

It has been argued that there are quality improvements that result from user charges [1-5], which reduce their negative impact on utilization, especially by the poor [6]. Although service quality has been hard to attain in many countries to a level that adequately compensates for the financial barrier set by user charges [7-10], many developing countries have maintained user charges however inequitable. The justification for this has largely revolved around the need to support additional investment in primary health care and maintain quality services, even when costs are subsidized in the medium term by international aid and/or concessionary loans [11].

In Uganda, following the introduction of user charges, evidence for improvements in quality of care was mixed. It was argued that the presence of user fees led to an improvement in the morale of the health workers, improved drug availability, and general maintenance of the health units [12], and there was some evidence suggestive of improvements in consumer-assessed quality of care [13]. This view was challenged by others [14], who argued that there was no evidence for improvements in technical quality of services that quality remained low even with the user fees. A national participatory poverty assessment [15] brought the cry of the population against the user fees to the policy table. As a result, the government abolished all forms of fees in all public health units on 1^st ^March 2001, with hospitals allowed to operate a paying window for those who could afford to pay. The policy aim was not to discourage payment for services, as prepayment mechanisms are not abolished, but rather to limit the financial barriers clients face in accessing health services by removing charges placed by government. In addition, fees continue to be charged in Private For Profit [PFP] and Private Not For Profit [PNFP] [predominantly church owned] facilities.

A number of measures were instituted to counter the expected effects of this policy change. There was an immediate release of US$ 526,315 [US$ 0.02 per capita] from the Finance Ministry to the districts to allow them purchase drugs. The total Government Primary Health Care [PHC] non-wage disbursement* to districts for the financial year [FY] 2000/01 was US$ 4,454,545, [US$ 0.19 per capita], with 50% recommended for drug expenditure^†^. Therefore, the additional release represented an increment of 22.3% on the expected drug expenditure by districts. As part of the budget process for the following financial year [starting 4 months after the policy change], there was an increase in the Ministry of Health allocations by US$ 12.5 million [0.52 US$ per capita]. This amount was felt to be adequate to compensate for the loss in revenue due to the policy change^‡^. The Primary Health Care [PHC] allocations to health units for recurrent expenditures increased. Non-wage allocations for the 2002/03 FY reflected a 165% and 66% increase for lower level health facilities and hospitals respectively on the 2000/01 financial year^§^. New guidelines were instituted for management of government grants, with increased flexibility to reflect the need to channel resources to areas previously supported by user fees revenue. A pull system for commodity supplies and management was also instituted. Wages for health workers were increased in the 2001/02 financial year by 14% – 63% across the different cadres of workers and in addition management of health workers pay roll was greatly improved, to ensure better human resources management.

This policy change provided the opportunity to study the effects of abrupt removal of direct costs of health services in Government facilities on quality of health services. We however note that there are indirect costs associated with seeking health care, but since the policy change was targeted at reducing direct costs, this study therefore focused on abolition of user fees, which is a direct cost, and its impact on quality of care. We studied how the different aspects of quality of care change as a country changes its health financing options away from user fees, with an aim to provide insights into policy actions countries could take to maintain quality of care immediately following the removal of formal user fees.

The public sector services are delivered in a Primary Health Care approach through a highly decentralized system, with the sub national units [districts] responsible for service delivery since 1996. The health system is further decentralized into functional health zones [Health Sub Districts, HSD], designed around the World Health Organization [WHO] Health District concept and functionally similar to the Close to Client system. At the time of the policy change [2001], there were 56 districts, and 214 HSD's. Services are provided through public, PFP, and PNFP facilities. The Health system is organized hierarchically, and is administratively managed at the national, regional and district levels. Service delivery is organized such that national and regional level facilities are equipped to respond to referrals from lower levels. At the district level, services are delivered at Health Sub Districts [HSD]**. HSDs are the implementation levels within a district and carry out planning, in-service training, coordinate service delivery and undertake supervision of lower level health units within their areas of responsibility. These HSD's are headed by HSD referral facility, which is a hospital or upgraded Health Centre IV.

The National referral hospitals provide comprehensive specialist services and, in addition, they are involved in teaching and research. Regional referral hospitals provide general curative and preventive services and specialist services. They provide technical supervision, on the average, to five districts. A general hospital provides general curative and preventive services, in-service training, consultation and research to community based health care programmes. A HC IV provides general preventive and curative services, emergency surgery and blood transfusion services. HC III and II, which are categorized lower level health units/facilities [LLU], provide mainly ambulatory services as included in the Uganda Minimum Health Care Package of services [UMCP].

The facilities providing these services are either Public, or PNFP facilities.

The policy change was targeted at the public facilities only. As there is close collaboration with, and direct support given to PNFP facilities at the time of the policy change, these too were assessed.

## Methods

Qualitative and quantitative research methods were applied. The effects of the policy change were investigated longitudinally over 36 months after the abolition user fees. Information was obtained on the 12 months preceding the policy change where feasible [data for 2000 was retrospectively reviewed]. The study took place between April 2001 and June 2004. Study sites were in 5 districts. These were selected purposively, with an aim to provide regional representation, the user fee scheme^††^; and varying poverty indices [PI]. The districts were: Rukungiri [western region; PI = 1.021] and Soroti [eastern region, with PI = 1.031] for districts had well-established schemes; Nebbi [northern region, PI = 1.047], Mubende [central region, PI = 1.022] and Rakai [central region, PI = 1.013] with ordinary user charges scheme. Poverty Indices in Uganda range from 0.894 for the least poor district which is Kampala in the central region to 1.055 for Kotido district which is the poorest in the northern region [16].

In each district, multistage random sampling was done within categories of health facilities, [public and PNFP] and the different levels of care to determine facilities, and sites for investigative follow up. First stage sampling was for selection of HSD referral facilities based on ownership [public/PNFP] and type [hospital/upgraded Health Center IV]. The targeted output was selection of an equal number [except for districts with limited numbers of PNFP referral facilities] of referral facilities from public, and PNFP sub sectors that were either hospitals or upgraded HC IV's. Second stage was for public and PNFP LLU within the areas of responsibility of the selected stage I facilities. The total number of health facilities was 85. Of these, 14 were referral facilities [11 public, 3 PNFP], and 71 were LLU [44 public, 27 PNFP]. From the catchment area of each selected LLU, one village with the highest utilization was selected in the first phase of the study amongst those with 30 or more new OPD attendants. Table 1 shows sampled health facilities.

**Table 1:** Sampled numbers and type of health units by district and ownership.

	**Public**						**PNFP [Private not for profit]**						
			
Type of HU	Mbde	Nebbi	Rakai	R'ri	Soroti	**S/T**	Mbde	Nebbi	Rakai	R'ri	Soroti	**S/T**	**Total**
Hospital	1	1	2	1	1	**6**	0	1	0	1	1	**3**	**9**
HC IV	1	1	1	1	1	**5**	0	0	0	0	0	**0**	**5**
HC III	4	3	1	3	8	**19**	6	3	5	5	2	**21**	**40**
HC II	2	12	4	6	1	**25**	0	0	1	4	1	**6**	**31**

**Total**	**8**	**17**	**8**	**11**	**11**	**55**	**6**	**4**	**6**	**10**	**4**	**30**	**85**

Each site had a study team of six people assigned to it. They visited their study sites from a central coordinating unit every 3 months starting 1 month after the policy change. Consistency of methodology within each study site was maintained by having the same team in the same site for the entire study period. It was felt that consistency in data collection would override any limitations relating to maintaining the same team over the study period.

Qualitative information was collected through Key Informant [KIs] interviews with key district and health facility officials representing the political, administrative and technical authorities respectively. A total of 140 KI interviews were conducted. A total of 71 Health Unit Management Committee members [HUMCs] at HC III and II were interviewed. Community views were sought through Focus Group Discussions [FGD's]. FGDs were conducted in public and PNFP facility catchment areas, which were determined by reviewing health facility registers and the village with the highest number of patients attending the facility was selected. FGD's, which comprised of a maximum of 12 participants each were conducted at each investigative phase. These were constituted by identifying the village chairman who provided a guide. Criteria for selection of participants were: knowledge of village issues, and representation of men, women, elderly, youth and disabled people. They must have lived in the village for the last five years and of voting age. A total of 71 FGDs were conducted. Information was collected on the therapeutic path followed by people when ill, with an attempt to track people's health care seeking behavior. Eight respondents were selected in identified villages. The starting point was the centre of the village, a pen was spinned to determine whether to start in the right or left direction. Once the direction was determined, four households were systematically selected and a questionnaire was administered to an adult [head of household or spouse] who was available and willing to be interviewed. After these four respondents, the team went back to the centre where they had started and systematical identified four households in the opposite direction. In instances where there was no adult, the next household was selected until the required number of respondents was met. We interviewed 587 persons from the communities we were investigating. Both users and non-users of facilities being investigated were interviewed, in villages served by public and by PNFP units. The purpose was not representativeness, but getting a synopsis of health seeking behavior among sampled communities.

Qualitative data was collected on the following variables:

1) *Health worker variables *[qualitative views on staff attitudes to work, their availability when needed, and staff motivation were sought through KIs and FGDs], and

2) *Client behavior in the absence of user charges *[changes in health seeking patterns among users and non users of health facilities.]

3) *Drugs and supplies: *Community and health worker's views [through KIs and FGDs] on drug availability and management were sought.

Quantitative data was collected from health records in all the sampled health facilities, to provide additional information on impact on quality of care. Proxy variables used to provide information on quality were:

1) *Drugs and supplies: *Data on quantities of drug receipts and average number of stock out days per month was collected from health facility stock cards. The two drugs most commonly used in outpatients, cotrimoxazole and chloroquine, were used as proxies.

The tools were pre-tested and translated into the local languages, and retranslated to English to ensure consistency. Transcription of qualitative data was done on the same day and data was entered into pre-designed matrix. Manifest content analysis technique was applied. Quantitative data was entered into pre-designed spreadsheets, with insignificant data entry error rates [<1%] and analysis done with significance tests done in STATA.

### Ethical consideration

The study was reviewed and approved by a committee constituted by the Ministry of Health department of planning and WHO country office Uganda. This committee also drew membership from the Ministry of Finance, planning and economic development and Makerere University School of Public Health, Department of Community Health and Behavioural Sciences. Permission was sought from the five district administration authorities as well as Community leaders to allow us carryout the study. Informed consent was obtained from the study participants after explaining the goals and objectives of the study, confidentiality safeguards and potential risks and benefits of the study were fully explained. The informed consent document was translated into the different local languages in the districts of study.

## Results

### Drugs and supplies

We followed trends in drug quantities from the drug stock cards at the health facilities. These are presented here as average [averaged for LLU and referral facilities] annual quantities received and average number of days in a month the drug is out of stock for the years of the study and; drugs in relation to number of OPD cases. The findings are presented for a period of 1 year prior to the policy change, up to 3 years after for the different levels of care [referral, and LLU's], and public and PNFP facilities.

**Figure 1:**
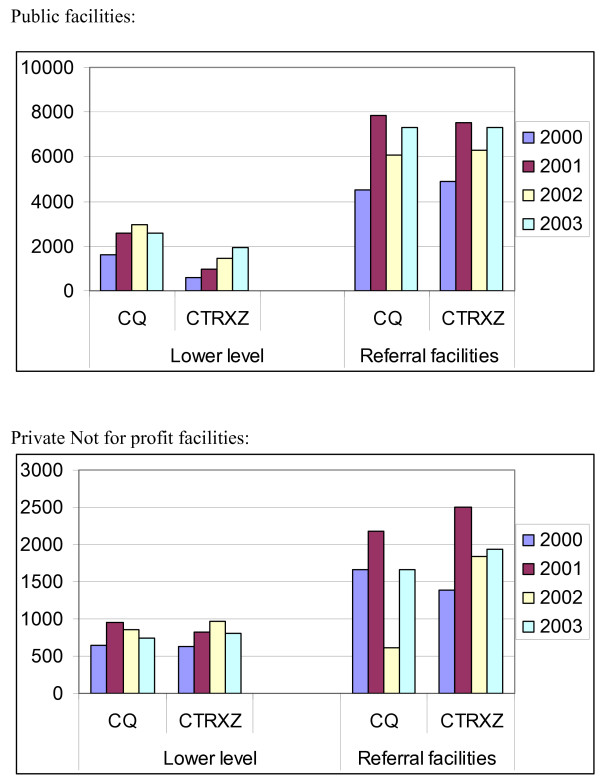
**Average annual drug receipts for Chloroquine and cotrimoxazole.** CQ: Chloroquine CTRXZ: Cotrimoxazole.

There was an increase in drug receipts following the abolition of the user fees in both LLU and referral public facilities as shown in Figure 1. No specific pattern in drug receipts was noted in PNFP facilities.

Regression Analysis was also undertaken, to determine if there was any significant change in drug availability after the abolition of cost sharing, in the various health care facilities.

Log(*Drug Availability*) = *f*(*Size, Ownership, Drug Category, Dummy For Abolition*)

Table 2 shows that results from both the OLS regression–where pooled drug availability is regressed on the size of the facility [i.e. whether referral or LLU, the ownership of the facility, the categorization of the drug in question and a dummy indicating the period when cost sharing was abolished, and the results from the pooled random effects model^‡‿^.

**Table 2:** Regression analysis to determine if there was any significant change in drug availability after the abolition of cost sharing:

Dependent Variable [Log of Drug Availability]		
Explanatory Variables	OLS Coefficient	Random Effects

Referral	-0.071	-0.0675
	[-1.01]	[-0.51]
Public	1.092***	1.085***
	[14.55]	[7.81]
Choroquine	-0.0257	-0.031
	[-0.37]	[-.045]
Dummy March 2001	-0.074	-0.074
	[-1.05]	[-1.07]
Constant	1.216	9.23
Number of Observations	2267	2267
Number of groups	-	32
Adjusted R-Squared	0.289	-
F(4,2262)	56.88	-
Prob >F	0	-
Wald Chi2(4)	-	66.8
Prob>chi	-	0

Notes: t values in parenthesis		

It was demonstrated that the indicator of abolition of cost sharing was not significant for both the OLS regression and the random effects model. The only significant correlates of drug availability were whether the facility is publicly owned. Specifically, the public variable [where government ownership is the default category] indicates that overtime; there was a significant increase in drugs availability in public health facilities as compared to PNFP facilities. This may be partly explained by the fact that it was public facilities that lacked the essential inputs prior to the abolition of costs sharing. Thus, the increased health spending was used to increase drug availability while in the PNFP facilities, the increased spending may have been used to pay for other recurrent costs.

Further investigation was done by looking at perceptions related to drug availability from the community. Almost all FGD respondents from both the public and PNFP units, throughout the study period, reported that health workers prescribe the needed drugs. FGDs in public facility catchment areas reporting that patients received prescribed drugs improved progressively during the study period. Forty four percent [44%] of HUMC and 60% of KI respondents also reported that drug availability was better in 2003 compared to 2001. In the PNFP catchment areas, majority of FGDs consistently reported that patients received prescribed drugs throughout the study period.

**Figure 2:**
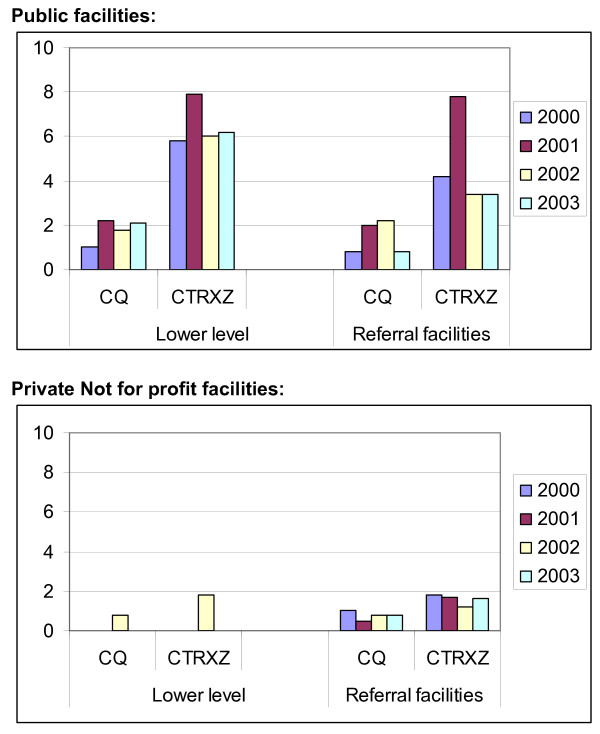
**Average monthly stock out days for facilities assessed for the period 2000 – 2003.** CQ: Chloroquine CTRXZ: Cotrimoxazole.

Average annual stocks out days were much higher in public facilities compared to PNFP facilities as shown in Figure 2. There was an increase in average annual stock out days in public facilities soon after abolition of cost sharing, which gradually reduced thereafter.

FGDs from half of public health facility catchment areas confirmed the finding that some patients do not get the prescribed drugs because they are not available. Twenty three percent [23%] of HUMC reported there were inadequate stocks of drugs although improvements in drug availability were noted in 2003 compared to 2001.

*"Drugs are brought here but not very frequently, the quantity delivered is inadequate, the patients are too many and so these drugs can not sustain them, especially the injectable drugs" *Member HUMC, public health facility June,2004.

When drugs were not provided at the health unit, the users reported employing a variety of coping mechanisms. The most commonly reported coping strategy was to visit private clinics or buy drugs from drug shops. This was reported both in areas served by both PNFP and public health units, and didn't change over the period investigated.

*"We get drugs when they are available and this has been rare since the abolition of user fee charges. We [now] normally purchase them from clinics or nearby drug shops. The poor of course go without drugs" *FGD, public health facility January 2001.

*We get the prescribed drugs if they are there. If they are not there, they tell us to go and buy from the private drug shops because they are out of stock and are still waiting for more stock to come" *FGD, public health facility June, 2004.

On the average, about 80% of the health unit staff from the public facilities reported not receiving drugs and supplies on time both before and after the abolition of user fees. For PNFP facilities, this was much less as only 12–16%; of PNFP facilities did not receive drugs on time over the study period.

*"We receive our drugs on time since purchasing is done by the dispensary. We have also been getting delegated funds, which contribute towards the purchase of drugs. We have no problem towards drug availability" *KI interview, PNFP facility, October 2001.

The reasons noted for non-receipt of drugs and supplies on time were further explored. District level bureaucracy was clearly a major problem hindering the availability of drugs and supplies at the public health units, and was increasingly reported over the year after the abolition of the user fees.

*"...we don't get drugs and supplies in time. Government takes so long to deliver these necessities but during the time we had cost sharing buying of drugs was easier because we had money at hand" *KI interview, public facility July 2001.

However, for the PNFP units, absence of funds is also quite prominently mentioned over the years. District bureaucracy was also noted, but was not as much a problem as in public units.

**Figure 3:**
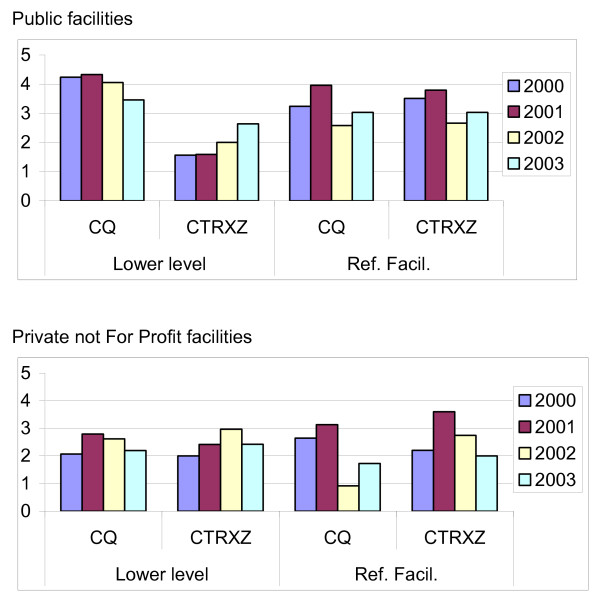
**Average monthly drug receipts/OPD case for the period 2000 – 2003.** CQ: Chloroquine CTRXZ: Cotrimoxazole.

The quantity of drugs prescribed per case remained fairly constant in both public and PNFP facilities except for cotrimoxazole in the Public lower levels where a slight increase is noted. This is shown in Figure 3.

### Health worker variables

Health worker behavior is an important component of quality health care provision. Qualitative information was sought from FGD's and KI interviews related to the health worker attitudes, motivation and availability. The majority of FGD's in public facility catchment areas reported that health workers were hardworking, good, and dedicated to their work consistently throughout the period assessed. This was also observed in the PNFP throughout the study period.

*"They are good staff, they advise us on the right drugs to use, the only problem is that the unit doesn't have drugs yet at the clinics we cant afford the charges" *FGD, public facility, July 2001.

A few negative responses made in public facility catchment areas included staff being too few, rude, not available when required, and were unqualified.

*"The health workers don't care. The one prescribing is slow, does his own things. You can go there at 9:00 am and leave at 2:00 pm [when] you end up giving up and go back home"* FGD, public facility, October 2001.

*"And they are also very rude, there is also segregation among the nurses, those who are known are treated properly and first, but those who are not known may end up mourning for the children" *FGD, Public facility, 2004.

The most common problems staff at the health unit reported when carrying out their duties were lack of transport, inadequate staff numbers, and poor support services like water, lighting, inadequate allowances and drugs. These made up 68% of all the responses, with the trend in reporting similar over the study period.

In public facilities, most of the staff had noted an increase in their income just after the abolition of user fees. However, the proportion of those with the view that their salaries had increased reduced over the study period. The main reason that could be attributed to the perceived reduction in income just after the abolition of user fees was a reduction in allowances they received.

*"I no longer participate in outreaches, hence no allowances for that. Completely no allowances in the last 3 months except for lunch allowances, no fuel to facilitate my movements"* KI Rakai hospital, Rakai district. July 2001.

However, in the later part of the study, salary delays were the prominent issue among the staff.

Staff motivational issues were further explored among HUMC members. About 50% of the HUMC's reported that staff were not motivated in any way. However, of those that reported some form of motivation, there was a significant drop [from close to 60% to below 10%] in reporting of allowances as means of motivating staff, with verbal appraisal and encouragement increasing [from close to 30% to over 50%] over the study period as the main method of staff motivation.

Verbal appraisal was less used as a motivational means in the PNFP facilities. The array of methods used to motivate the staff in PNFP facilities was wide. These included ensuring staff are paid timely, offering free treatment to staff and allowing them to work elsewhere such as teaching during their free periods.

### Patient health care seeking behavior in the absence of user charges

During the fourth investigative phase [12 months after the policy change], we studied the therapeutic path patients take when seeking care in the absence of user charges. This was aimed at exploring the numbers of health care options people in the community use and the average time spent at each option. Both users and non-users of the public, and PNFP health facilities were interviewed. It was demonstrated that, health care seeking options were numerous. However, during analysis, health care seeking options were stratified into public or PNFP facilities, private clinics, drug shops and traditional providers.

Of all the respondents, 285 and 136 persons from areas served by public and PNFP facilities respectively reported being users of these facilities, while 86 and 80 reported not using them. The majority [90%] reported favorable final illness outcomes from care sought in areas served by both public, and PNFP facilities.

**Figure 4:**
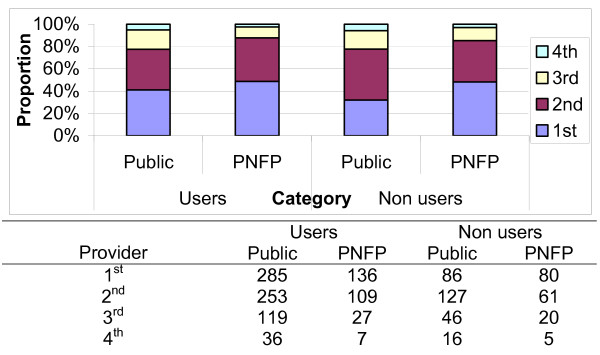
Number of providers seen by communities in different categories of providers.

The number of providers seen in order to achieve the illness outcomes they had by the different categories was reviewed and is shown if Figure 4. More than 80% of both the users and the non-users of the facilities had achieved their illness outcomes after use of 2 providers. A higher proportion of both users and non-users in villages covered by PNFP facilities achieved their illness outcomes before those in areas covered by public facilities. This implies there were more persons using 3 to 4 providers among the users and non-users in villages with public facilities as opposed to those in PNFP facilities.

Among the people that reported not using the health facilities under investigation, we reviewed the providers of choice. Private clinics were the most used health care option among those not seeking care from the public facilities [41%]. These were however only used by 17% for the PNFP facilities among whom the more common health care providers were tertiary care providers.

Finally, we reviewed the time spent with each provider. The respondents were asked how long they spent with each provider they went to, before choosing to move on to the next provider.

**Figure 5:**
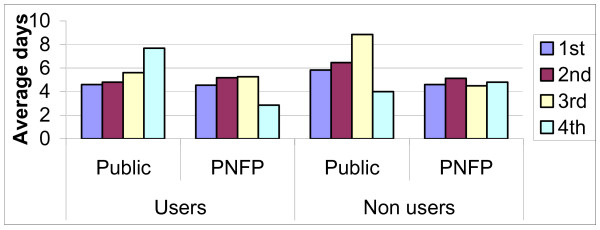
Average time spent with each provider in the different catchment areas.

The average time spent at each provider increases with the number of providers seen as shown in Figure 5. The persons that spend the longest time were those who used traditional herbs as the first treatment option, while the least time was spent on self-treatment.

Regarding utilization of health facilities, there were marked in increases in utilization in public facilities and slight increases were also noted in PNFP facilities. Percapita utilization of Out Patients Department in government and PNFP facilities increased from 0.43 in 2000, to 0.6 in 2001 to 0.8 in 2004 [17].

## Discussion

We have reviewed some aspects of quality of care that are likely to provide insights into overall quality changes following the abolition of user charges in health facilities in a developing country setting. There are two aspects of quality of care in any health system; observed [technical] and perceived/consumer assessed [6]. Technical quality of care relates to defined standards of care, while consumer assessed quality of care relates to the views of users/potential users of health services. There is extensive evidence that shows users perceptions of quality of care have a strong influence on utilization [1,2]. Technical quality insights are provided by staff characteristics and drug availability issues from review of records and information from community interviews.

### Technical aspects of quality

The findings show that although drug receipts were improved after the abolition of user charges, community views showed that many patients were not able to obtain their prescriptions. However, a small number of people in the communities were able to. As this was the similar situation before the policy change, we can say that in spite of charging communities fees, they felt few were receiving drugs. This situation is similar to what had been observed in other areas where the ability of user fees to effect meaningful quality improvements have been challenged [10,11,14]. The improvement in drug availability seen at the LLU is attributed to increased PHC allocation to these facilities and the requirement for at least 50% of these PHC recurrent non wage resources to be spent on drug purchases.

It was observed that the general view of the public was that there was no availability of drugs in the health centers. Although this view is contrary to the actual data obtained since there was an increase in drug receipts, it should be noted that there was an increase in visits to the health centre resulting in proportionately fewer people receiving their drugs and an increase in the number of disgruntled clients. The observations from this study does not also support the fact that there was rationing of drug by the health care workers as there was no reduction in drugs per/OPD case.

With user fees, health facilities were able to make drug purchases outside the public system that were based on their requirements. The policy change implied a shift to predominantly purchase through the public system with a resultant reduction in flexibility and unnecessary delays. This is seen by the high reporting of district bureaucracy as the main cause of delays in getting supplies to the health facilities.

During the era of user fees, many support staff and technical staff were receiving wages [for those who were not on the public service payroll] and or allowances from user fee revenues. At the abolition of user fees, many of the support staff had to be laid off and in many cases the income of some of the technical staff dropped. This resulted in human resource crisis in some of the centers, as the limited staff had to deal with increased load of patients since the services and especially the drugs were now free to the population. To mitigate against this, there was an attempt at accelerated recruitment of health workers by the centers. The effect of the accelerated recruitment of health workers was not easily discernable due to lack of credible information on health work force data before and after the policy change. However, indirect evidence [high community satisfaction with health workers, and reducing disgruntlement among them] suggests two situations. The health workers either adapted to the increased workload favorably, or the increased recruitment was adequate to cover the increased needs due to the higher client load.

Health workers attitudes were poorer in the public health units as opposed to the PNFP units possibly as result of a poorer incentive structure. The policy change had the potential to even make this worse. Presence of use fees implied that health workers had resources available for use when required, as opposed to the public resources that have stringent guidelines for spending and accountability attached to them. The government response to the abolition of user fees included an increase in resources to the health workers, at levels much better than before the abolition of user fees. Continued health worker disgruntlements after the pay increases were therefore due to two possibilities. The loss of a source of resources that were readily available as needed [a situation promoted by user charges, which are ever there when needed] or the amounts of fees that were being collected were significantly more than was being reported, and so there was a real decrease in the health workers income that wasn't compensated for adequately by the pay increases.

When the present health care seeking patterns of communities is examined, it was observed that the inability to satisfy clients was not a characteristic of the public facilities only. This is because, all the significant health care providers presently offering services to the communities are not able to holistically manage the medical needs of their clients. Thus, the clients have adjusted their expectations in line with the services available from the different providers. This implies that, from the consumer's point of view, there is wholesome low quality of care among all providers of care, not just the public facilities. The presence of user charges [which all the other care providers still employ] does not imply there will be improved care quality from the consumers' viewpoint.

## Conclusion

From these findings, we can conclude that the levels of technical quality of care attained in a system with user fees are not necessarily a result of the user fee policy, as this quality can be maintained, or even improved without the fees. Such quality can be achieved through adoption of basic system modifications, coupled with marginal increases in funding that are within the reach of developing countries. This is based on targeted increases in allocation to health, with the focus on ensuring more resources are available at the implementation level, and a systemic change in the way in which health services are managed particularly regarding flexibility in the use of public funds at the service delivery level to allow them adjust the required inputs in line with locally determined requirements. The initial response should commence before the actual policy change. Other mechanisms for providing financing to the financing intermediaries that would strengthen quality of care can also be explored, such as Output Based Aid approaches.

The policy change was aimed at limiting financial constraints to users, but is not the only option available for countries. These other financing options, such as community based insurance schemes have the potential to achieve the similar aims, if they are able to have equitable involvement of vulnerable persons. Many of the schemes in Uganda operating through public facilities had to shut down, as the policy change was able to provide most of the benefits they were providing, but in a more equitable and efficient manner.

However, the residual implications of perceived reduction in quality, particularly relating to the reported reduced motivation, and a potential for increase in unofficial payments among health workers illustrates the residual implications of such a policy change. A trade-off between these residual effects, and the welfare gains by the population should guide move towards such a policy change by a country. Further analytical work is needed to quantify the impacts of these options in more detail.

Finally, we conclude by stating that the management of quality in any system is a sub function of the overall management process. As such, improvements in management of quality are usually heavily driven by improvements in overall management. This was seen in that the responses to the policy change that led to quality improvements were designed to also lead to overall improvements in management of services in a holistic manner. Targeted and patchy changes focusing only on quality issues may not lead to the same outputs as we have demonstrated. What are needed are comprehensive changes across different facets of management of health services to maintain quality.

## Competing interests

The authors declare that they have no competing interests.

## Authors' contributions

JN-O participated in conceptualization of the study, data collection and analysis and, led the drafting of the manuscript; HK participated in conceptualization of the study, data collection, analysis and drafting of the manuscript; LA participated in conceptualization of the study, data collection and analysis and, drafting of the manuscript; FB participated in data collection, analysis and reviewed the manuscript; SAO participated in data analysis and reviewed the manuscript, OW participated in conceptualization of the study, data analysis and writing of the manuscript.

## Note

* Public resources are disbursed in form of grants to different intermediaries

^† ^Guidelines for utilization of PHC conditional grants; MoH; 1998.

^‡ ^Background to the budget MoFPED; 2001/02

^§ ^Background to the budget MoFPED; 2002/03

** A HSD, which is headed by a hospital or an upgraded HC IV and located at a country level, serves an average population of 100,000 population. It is a network of community-based health centers, which provides support to the lower-level health centers and manages referral cases. A HC III is located at a Sub country level and serves an average population of 50,000. A HC II is located at a parish level and serves an average population of 25,000.

^†† ^Some districts in the country were implementing a fee-for-service scheme [Bamako initiative type of cost recovery], some had normal user fees, while a few had not/were just starting to implement any form of cost sharing.

^‡‡ ^We also tried estimating the fixed effects model which unfortunately was dropping two key variables size and ownership of the facility.

## Pre-publication history

The pre-publication history for this paper can be accessed here:


